# Finding the masseteric nerve: an illustrated surgical guide for facial reanimation

**DOI:** 10.1016/j.jpra.2026.07.042

**Published:** 2026-08-13

**Authors:** T. Stigger, K. Ingels, N. van Heerbeek, G. Damen, A. Scheffer, EM. Morandi, D. Ulrich, R. Magnato, B. Kofler

**Affiliations:** a Department of Plastic, Reconstructive and Aesthetic Surgery, Medical University of Innsbruck, Innsbruck, Austria; b Department of Otolaryngology Head & Neck Surgery, Radboud University Medical Center Nijmegen, Nijmegen, the Netherlands; c Division of Otorhinolaryngology & Maxillofacial Surgery, Franz Tappeiner Hospital, Meran-o, Italy

**Keywords:** Facial palsy, Facial reanimation, Nerve transfer, Masseteric nerve, Reconstructive surgery

## Abstract

**Background:**

The masseteric nerve (MN) is a reliable donor nerve in facial reanimation. Masseteric-to-facial nerve transfer offers advantages including close proximity to facial nerve branches, strong axonal input, and minimal donor-site morbidity. However, safe and efficient identification of the MN can be challenging, particularly for surgeons less familiar with this region. A structured, reproducible approach is therefore essential.

**Methods:**

14 fresh-frozen hemifaces were dissected to identify the MN and define its topographic relationships. Distances were measured relative to the apex of the tragus, the caudal edge of the zygomatic arch, and the superficial musculoaponeurotic system (SMAS). Based on these findings, a step-by-step surgical guide was developed, completed by schematic illustrations and an intraoperative video tutorial.

**Results:**

The MN was consistently located 3.89 ± 0.27 cm anterior to the apex of the tragus, 1.01 ± 0.18 cm inferior to the zygomatic arch, and 1.11 ± 0.21 cm deep to the SMAS. It was found in close proximity to the frontal branch of the facial nerve, typically directly beneath it or slightly anterior or posterior to it. Despite minor anatomical variations, a reproducible dissection pathway was defined. The resulting illustrated guide and video tutorial highlight key anatomical landmarks and safe dissection zones.

**Conclusions:**

This cadaveric study and surgical tutorial provide a practical, reliable guide to identify the MN. The structured, step-by-step approach is designed to support both trainees and experienced surgeons, thereby improving safety, precision, and surgical efficiency in facial reanimation.

## Introduction

Dynamic reanimation is the preferred surgical technique in facial reanimation of the lower third, offering both functional and aesthetic benefits. Standardized surgical techniques have been established for dynamic smile restoration, with the masseteric nerve (MN) among the most commonly used nerves in these procedures.^1^

The MN arises from the anterior division of the mandibular branch of the trigeminal nerve and represents its largest motor branch.^2^ After exiting the skull via the foramen ovale, it courses over the lateral pterygoid muscle. It traverses the coronoid notch, just anterior to the mandibular condyle and posterior to the temporalis muscle insertion,^3^ before entering the posterior surface of the masseter muscle near its origin. The masseteric muscle is typically innervated by multiple branches.^4^

Spira first introduced the use of the MN in facial reanimation through coaptation of the MN with a nearby lower division of the facial nerve (masseteric-to-facial nerve coaptation, MFC) in 1978.^5^ Later, the MFC was used in the so-called “babysitter procedure” to maintain muscle tone on the paralyzed side while the cross-facial nerve graft (CFNG) reached the motor endplate of the affected side.^6^ The MFC was subsequently applied in facial reanimation surgery as a permanent procedure^7^ and is today a reliable and versatile method, performed either as a single-stage procedure or in combination with other dynamic or static techniques.^1^ Sforza et al. described the MFC as a powerful component of the triple innervation, together with CFNG and hypoglossal-to-facial nerve coaptation, in facial reanimation.^8^ Another function of the MN in facial reanimation is to supercharge preexisting facial nerve coaptation, thereby providing greater power.^9^

Several advantages made the MN one of the most essential sources for dynamic facial reanimation. One of the major advantages is rapid reinnervation of the facial muscles, typically within 3–4 months. The anatomical proximity to the facial nerve allows tension-free end-to-end coaptation. In addition, the MN contains a high number of motor fibres, resulting in a strong excursion of the oral commissure.^4^^,^^10^^,^^11^ Functional deficits from harvesting the MN are minimal, as the masseteric muscle is typically redundantly innervated by two or more masseteric nerve branches. In addition, compensatory activity of the temporalis and pterygoid muscles helps preserve masticatory function despite partial denervation.^4^^,^^12^ Despite its advantages and widespread use, intraoperative identification of the MN remains challenging, particularly for less experienced surgeons.

Borschel et al. described the MN as consistently located 3.16 ± 0.30 cm anterior to the tragus, 1.08 ± 0.18 cm inferior to the zygomatic arch, and 1.48 ± 0.19 cm deep to the superficial musculoaponeurotic system (SMAS).^12^ In 2013, Collar et al. proposed the subzygomatic triangle to identify the MN, defined by the zygomatic arch superiorly, the temporomandibular joint (TMJ) posteriorly, and the frontal branch of the facial nerve inferiorly and anteriorly.^13^ Cheng et al. presented a technique to identify the nerve within a triangular area measuring 1.5 cm^2^, defined by constant anatomical landmarks – the zygomatic arch, condyle, coronoid process, and mandibular notch.^14^ Other studies described the MN to lie on an artificial axis between the TMJ and the chin tip, with an average distance of 3.5 cm from the TMJ.^15^

While these anatomical definitions are helpful, intraoperative identification of the MN remains difficult. The objective of this study is to develop a visual and video-based step-by-step surgical guide for reliably locating the MN using reproducible anatomical landmarks.

## Materials and methods

### Study design

This anatomical study was conducted at the Department of Anatomy, Radboud University Medical Center, Nijmegen, The Netherlands. Human cadavers were obtained through the Dutch body donation program for science and education in accordance with national legislation (Wet op de lijkbezorging, Art. 18 §1, Art. 19, Art. 67–70, April 1991, w.wetten.overheid.nl/BWBR0005009). Body donations from individuals aged 18 years and older with a valid handwritten statement of consent were accepted.

### Specimens and anatomical dissection

For dissection, 14 hemifaces from ten fresh-frozen adult cadavers were used. Five were male, five were female. Nine cadavers were Caucasians, and one was African-American. Predefined landmarks for MN localization were the apex of the tragus, the caudal edge of the zygomatic arch, the depth to the SMAS, and the topographic relationship to the frontal branches of the facial nerve. The results were documented with photographic imaging.

A facelift-type incision was performed, and the skin flap was elevated. The parotid fascia was exposed, and in the sub-SMAS plane, along the anterior border of the parotid gland, the branches of the facial nerve were identified and dissected. Dissection was continued anteriorly to the anterior border of the masseter muscle.

To identify the MN, the superior portion of the masseter muscle was gently detached from the zygomatic arch. Blunt separation of the muscle fibers along their natural fiber direction was performed using two small Langenbeck retractors, progressing until the deeper portion of the masseter muscle was exposed and the MN became visible. When needed, the mandibular notch was palpated to assist localization, as the MN emerges from this landmark.

### Measurements and statistics

The distance from the MN to the reference landmarks was measured using a standard metric ruler. Descriptive statistics were calculated with means and standard deviations. Sex-based differences were analyzed using the Mann-Whitney U test due to the small sample size. Data are presented as median and interquartile range (IQR), with exact two-tailed p-values reported and statistical significance set at p < 0.05. Statistical analyses were performed using Microsoft Excel (Microsoft Corp., Redmond, WA, USA) and IBM SPSS Statistics (IBM Corp., Armonk, NY, USA).

### Illustrations & video

Illustrations were created in Procreate (Apple Inc., Cupertino, CA, USA) on an iPad, with additional rendering support provided by the online platform Illustrae. The intraoperative video was recorded during a facial reanimation procedure. Written informed consent of the patient was obtained.

## Results

### Anatomical findings

**Fig. 1:**
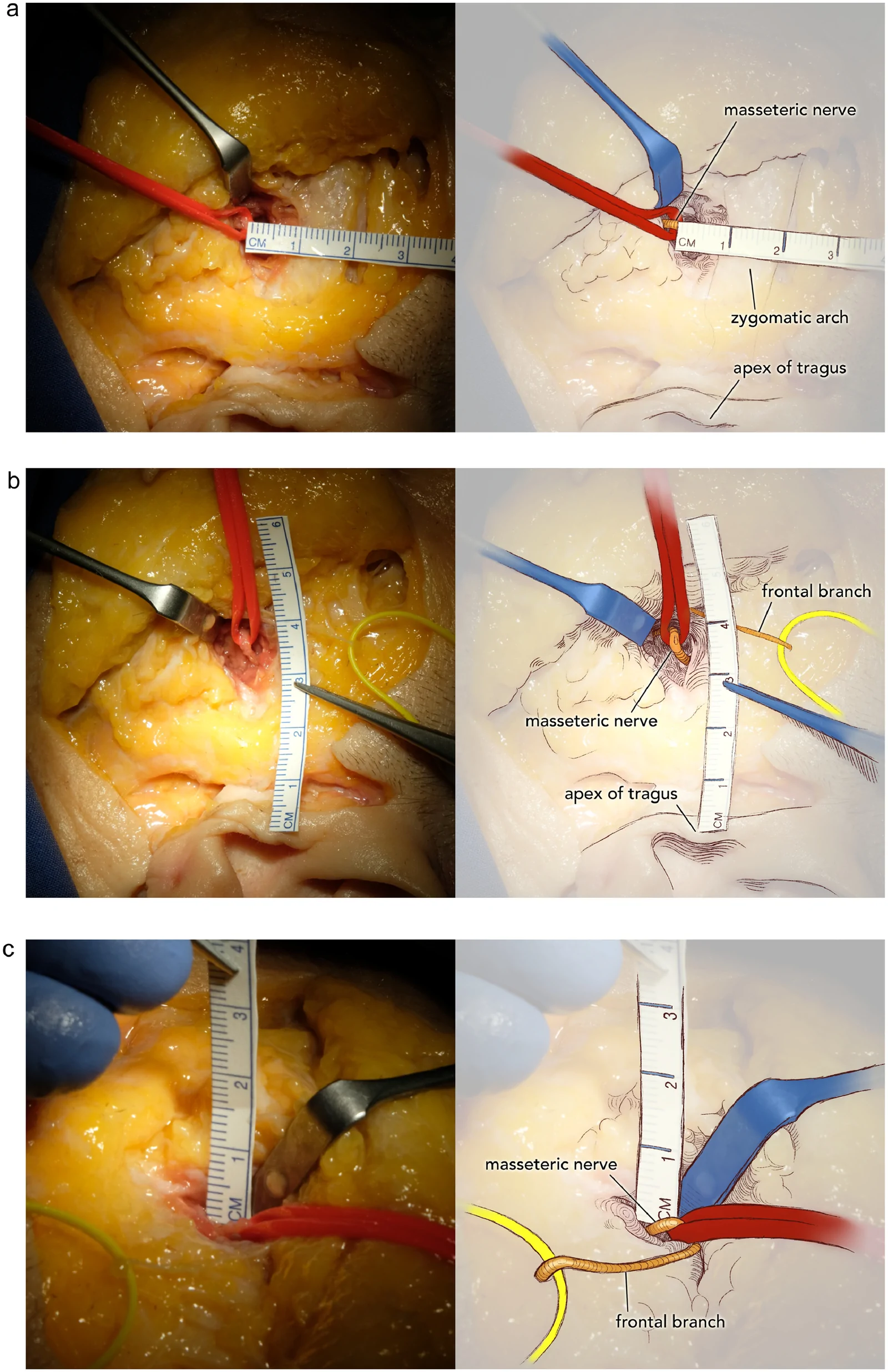
Measurements of the masseteric nerve on the left hemiface: a) distance from the inferior border of the zygomatic arch, b) distance from the apex of the tragus, c) depth to SMAS (view from superior). The frontal branch of the facial nerve is gently retracted anteriorly with a small Langenbeck retractor, exposing the MN immediately beneath or adjacent to it.

Four reliable anatomical reference points in relation to the MN were identified: the apex of the tragus, the caudal edge of the zygomatic arch, the SMAS, and the frontal branch of the facial nerve. The MN was located 3.89 ± 0.27 cm anterior to the apex of the tragus and 1.01 ± 0.18 cm inferior to the caudal edge of the zygomatic arch (Fig. 1a,b). The MN was found at a depth of 1.11 ± 0.21 cm to the SMAS (Fig. 1c). In all specimens, the MN was located in close proximity to the frontal branch of the facial nerve, typically directly beneath it or slightly anterior or posterior to it (Fig. 1b, c).

No statistically significant sex differences were observed in MN location. The median distance from the tragus to the MN was 3.85 cm in males versus 3.75 cm in females (Mann-Whitney U = 23, p = 0.897); the zygomatic arch distance measured 1.00 cm in both groups (Mann-Whitney U = 24, p = 1.000); and MN depth relative to the SMAS was 1.20 cm in males versus 1.00 cm in females (Mann-Whitney U = 14, p = 0.197). All measurements are summarized in Table 1.

**Table 1:** Location of the masseteric nerve (MN) relative to anatomical landmarks.

Measurements	Overall (n = 14) Mean ± SD (cm)	Male (n = 8) Median (IQR, cm)	Female (n = 6) Median (IQR, cm)	P-Value (Male vs. Female)*
Tragus-to-MN Distance	**3.89±0.27**	3.85 (3.70–4.03)	3.75 (3.70–4.03)	0.897
Zygomatic arch-to-MN Distance	**1.01±0.18**	1.00 (1.00–1.05)	1.00 (1.00–1.00)	1.000
Depth SMAS-to-MN	**1.11±0.21**	1.20 (0.98–1.40)	1.00 (0.93–1.08)	0.197

*P-Values refer to comparisons between male and female specimens.

### Operative approach

The procedure is performed under general anaesthesia. Neuromuscular blockage should be avoided, as intraoperative nerve stimulation is essential for accurate identification of the MN.

1. **Preoperative Markings** (Fig. 2a).
  − Mark a facelift-type preauricular incision, extending cranially in a zig-zag, trichophytic pattern into the temporal region and inferiorly along a standard cervicofacial extension as required.
  − Mark the inferior border of the zygomatic arch.
  − Mark the expected course of the frontal branch along the Pitanguy line.^16^^,^^17^
  − Mark the expected location of the MN 4 cm anterior to the apex of the tragus and 1 cm inferior to the inferior border of the zygomatic arch.

**Fig. 2:**
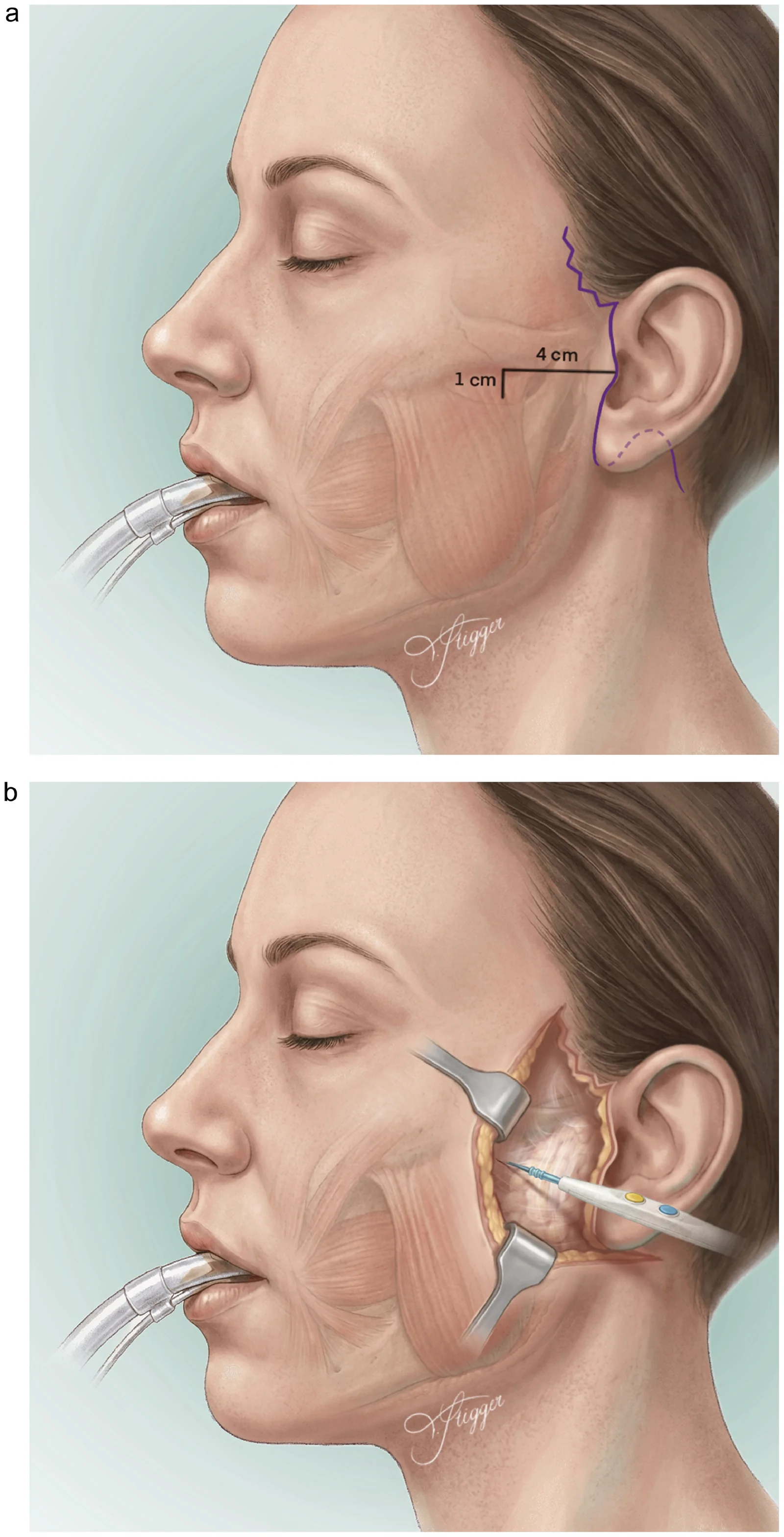

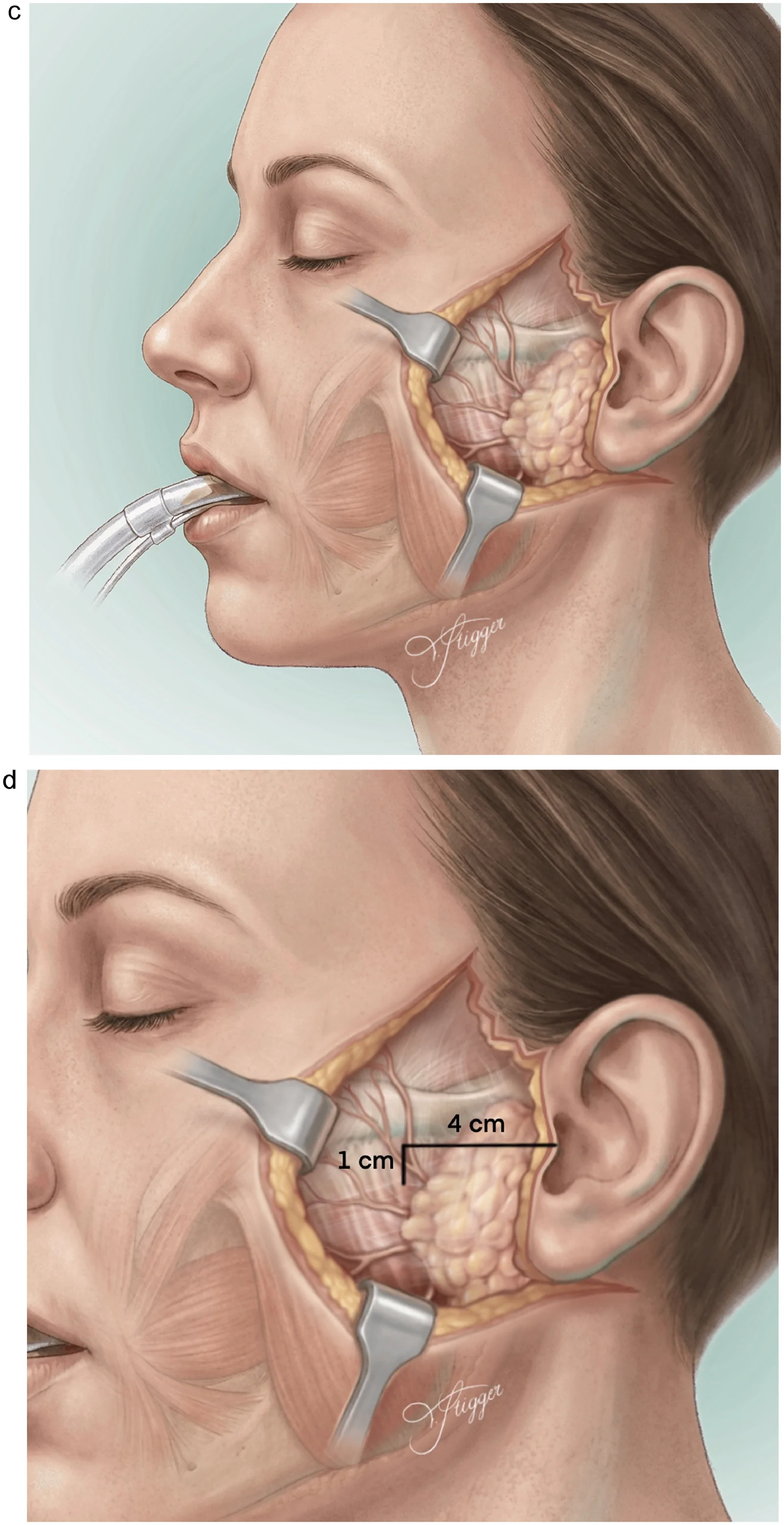

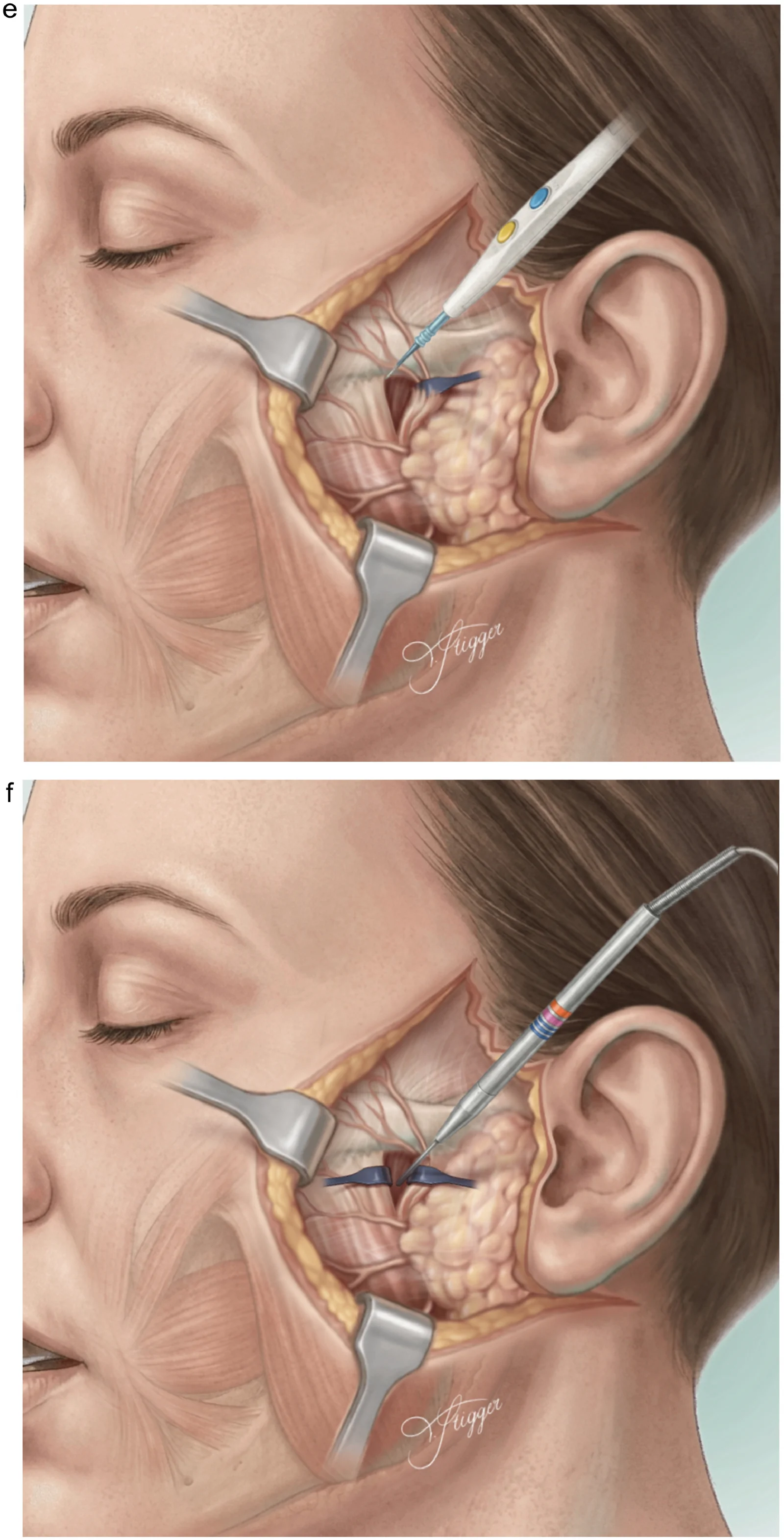

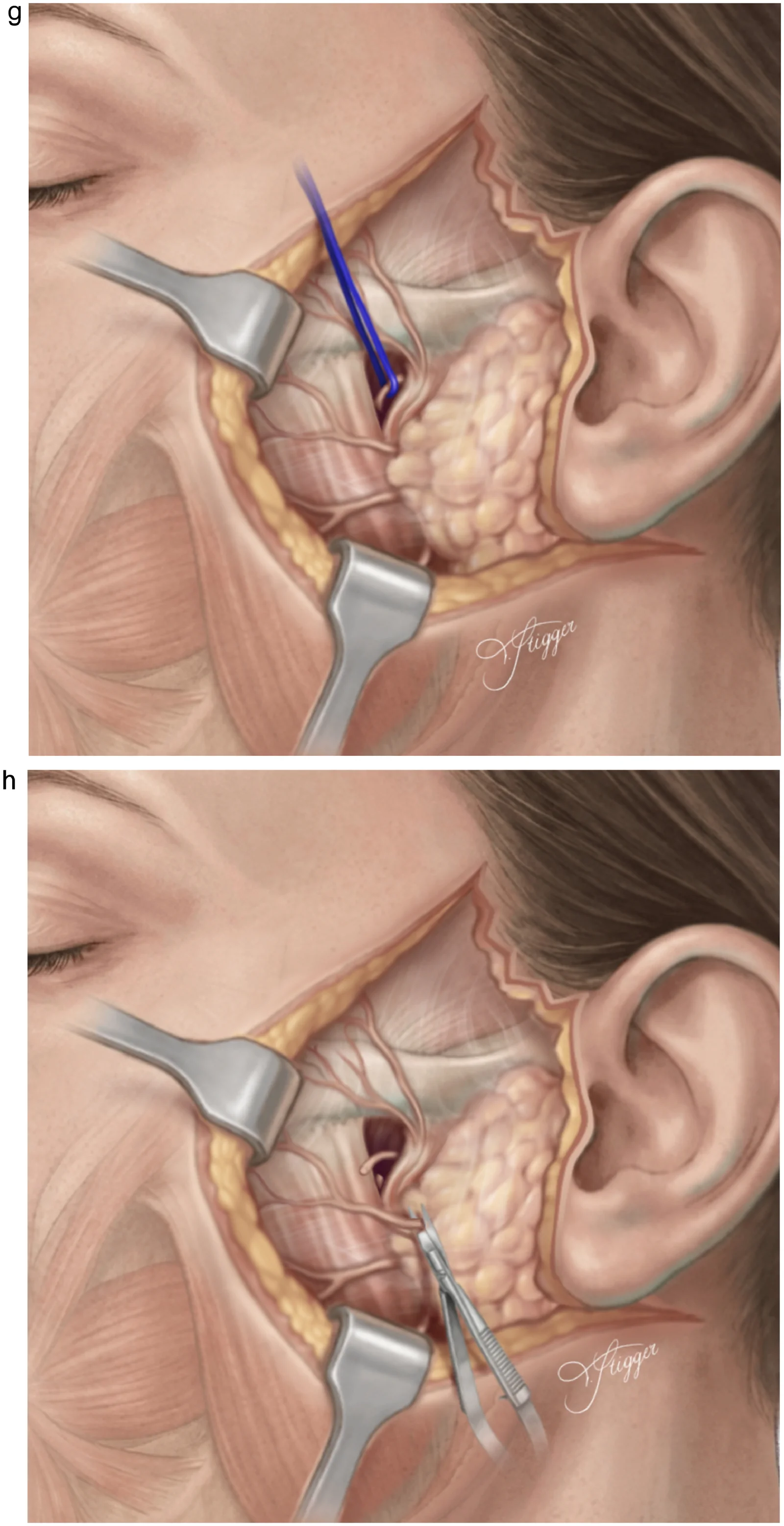

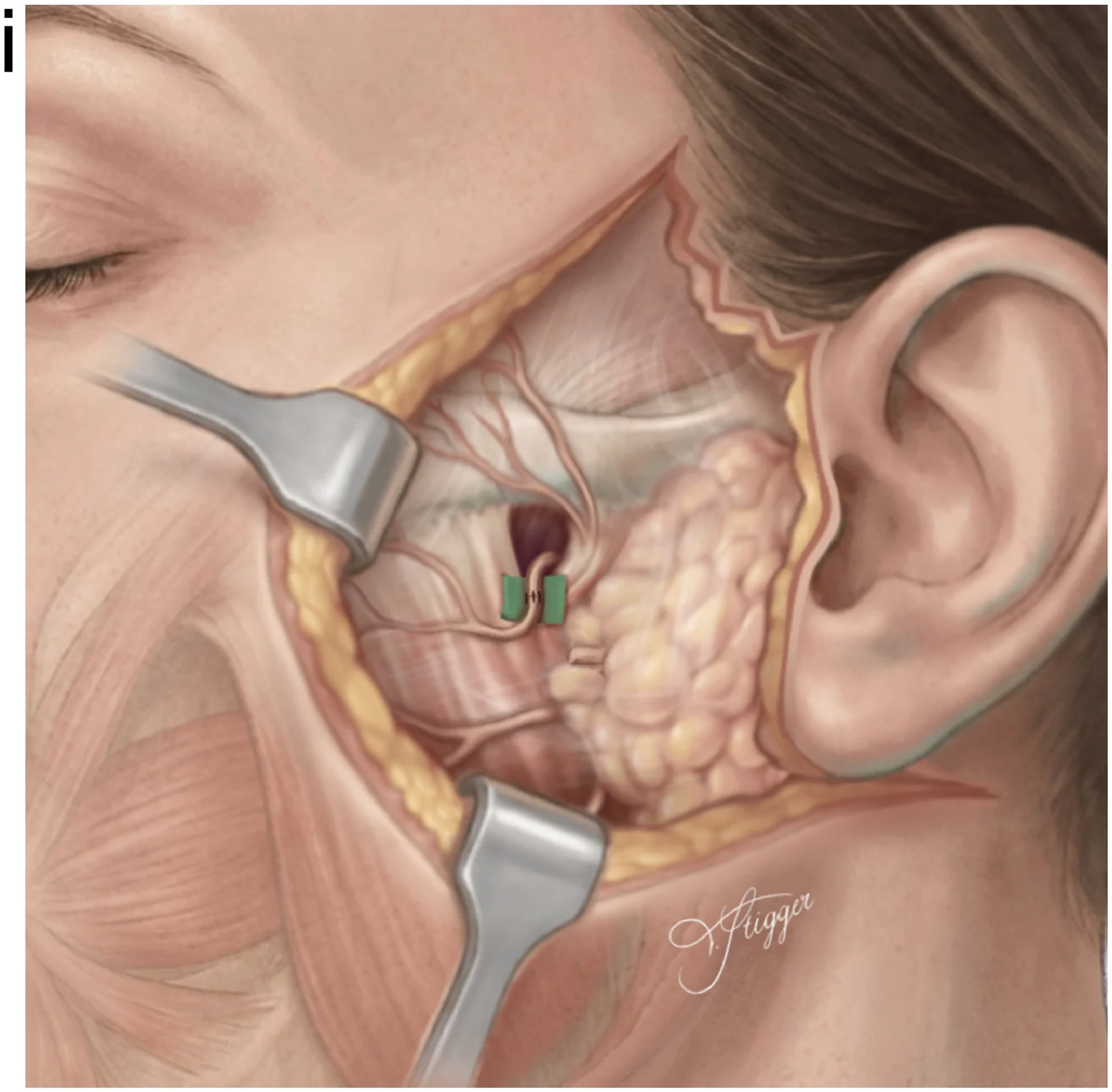
Operative approach: a) preoperative markings, b) skin flap elevation, c) identification and preparation of the facial nerve branches, d) identification of the MN: measurements, e) identification of the MN: detachment of the superior fibers of the masseteric muscle from the zygomatic arch, f) identification of the MN: blunt dissection and use of nerve stimulator, g) MN marked with vessel loop, h) transection of MN and zygomatic branch: transect the MN as far distally and the facial nerve branch as far proximally as possible to allow tension-free coaptation i) coaptation of the MN and facial nerve branch using 10–0 nylon sutures under an operative microscope.
2. **Local Infiltration and Skin Incision.**
  − Infiltrate along the incision line with adrenaline-containing saline solution (e.g., 1:200.000 epinephrine in 0,9% NaCl) for hemostasis.
  − Perform the skin incision along the marked facelift-type line using a No. 15 blade scalpel.
3. **Skin Flap Elevation (**Fig. 2**b).**
  − Elevate the skin flap in a subcutaneous plane using monopolar electrocautery.
  − Maintain a uniform thickness to preserve perfusion and reduce postoperative contour irregularities.
  − Continue dissection until the parotid fascia is clearly visualized.
  − Dissect anteriorly along the parotid fascia until the anterior border of the parotid gland is exposed.
4. **Identification and Preparation of the Facial Nerve Branches (**Fig. 2**c).**
  − Along the anterior border of the parotid gland, transition from the subcutaneous to the sub-SMAS plane. Then, working in a sub-SMAS layer, identify the zygomatic and buccal branches of the facial nerve using loupe magnification.
  − Perform blunt, parallel dissection with fine scissors to avoid traction or transection injury.
  − Mark identified branches with vessel loops.
  − Select the branch (zygomatic or buccal) with the largest caliber appropriate for masseteric coaptation (typically 1.0–1.5 mm; adapt to your final measured value after identification of the masseteric nerve branch).
  − If required, dissect the selected branch retrogradely into the parotid tissue until a healthy, tension-free segment is available for coaptation.
5. **Identification of the Masseteric Nerve (**Fig. 2**d-g).**
  − Confirm the surface marking of the expected MN location, approximately 4 cm anterior to the apex of the tragus and 1 cm interior to the inferior border of the zygomatic arch.
  − The frontal branch of the facial nerve is typically found in close proximity to this region and can serve as an important anatomical guide, as the MN lies immediately deep and adjacent to it.
  − Detach the most superficial fibers of the superior portion of the masseteric muscle from the zygomatic arch to facilitate exposure.
  − Insert two small Langenbeck retractors and gently split the muscle fibers in a blunt, vertical direction parallel to the muscle fibers.
  − Proceed until reaching the deep portion of the masseter muscle.
  − The expected depth of the MN is approximately 1.1 cm below the SMAS.
  − Use a nerve stimulator to identify MN branches.
  − The mandibular notch may be palpated to guide identification. To facilitate palpation, the mandible can be moved during surgery.
6. **Nerve Division (**Fig. 2**h).**
  − Transect the masseteric nerve distally to obtain maximum usable length.
  − Transect the selected buccal or zygomatic branch proximally, ensuring adequate length for tension-free alignment.
7. **Nerve Coaptation (**Fig. 2**i).**
  − Under the operating microscope, perform a precise end-to-end epineural coaptation using a minimal amount of 9–0 or 10–0 nylon sutures.
  − Ensure proper fascicular alignment and absence of tension.
  − Apply a small amount of fibrin glue to reinforce the coaptation site and minimize micromotion.
8. **Hemostasis and Closure.**
  − Perform meticulous hemostasis.
  − Reapproximate tissue in layers and close the skin following standard facelift closure principles.

**Video 1)** Step-by-step surgical identification of the masseteric nerve.

## Discussion

The MFC has become a widely accepted and reliable technique for dynamic facial reanimation over the past decade, providing robust, rapid reinnervation with high rates of smile recovery. However, intraoperative localization of the MN remains challenging due to a lack of precisely defined anatomic landmarks and its deep intramuscular course within the masseter. Prolonged nerve identification may increase operative time, bleeding, and the risk of iatrogenic injury.^18^ Establishing precise and reproducible anatomical reference points is therefore crucial, particularly for surgeons early in their learning curve and in cases requiring staged procedures.^19^

The most commonly cited method for MN localization is described by Borschel et al.^12^ However, their measurement points along the tragus and zygomatic arch were not explicitly defined, limiting reproducibility. In this study, we defined the apex of the tragus and the caudal edge of the zygomatic arch as more precise, consistent, and easily identifiable reference points. Because the tragus is relatively mobile and may be displaced during retraction, surgeons should regularly reassess its position.

Our measured distances align closely with those reported in previous studies. Regarding the distance from the MN to the zygomatic arch, our result (1.01 cm) is consistent with that of Borschel et al. (1.08 cm). Compared with the tragus, our mean distance was 3.89 cm anterior, whereas Borschel et al. reported 3.16 cm,^12^ likely because their measurements were taken from the anterior aspect of the tragus. Abdelkarim et al. described a similar tragus-to-MN distance (4.003 cm) to that in this study and noted sex-related differences, with men demonstrating a greater distance to the zygomatic arch and a positive correlation between zygomatic arch-to-MN distance and intramuscular depth.^13^ In our cohort, male specimens tended to have a greater tragus-to-MN distance and nerve depth; however, these differences did not reach statistical significance, likely due to limited sample size.

In comparison to the subzygomatic triangle as described by Collar et al.^13^ – bounded by the zygomatic arch, the TMJ, and the frontal branch of the facial nerve - we also observed the MN anterior to the frontal branch. This suggests that the subzygomatic triangle may not reliably encompass the MN across all anatomical variations, underscoring the value of linear, reproducible measurements to improve precision in nerve localization.

The relationship between the MN and the frontal branch of the facial nerve is variable and should be considered carefully during dissection. In our observations, the MN was located either directly beneath or immediately anterior or posterior to one or more small branches of the frontal nerve. Blunt retraction of the frontal branches allows safe exposure, and once any frontal branch is identified, the MN can be reliably found immediately deeper in the same region.

Based on our anatomic findings, we recommend the apex of the tragus and the caudal edge of the zygomatic arch as reliable surface landmarks for MN localization. Surgeons should expect the MN to lie way deeper than anticipated, on average 1,11 cm beneath the SMAS. Therefore, we advocate for initial detachment of the most superficial fibers of the superior portion of the masseter muscle from the zygomatic arch, after which the muscle is split along the direction of its fibers using Langenbeck retractors. Once the deep portion of the masseter muscle is reached, the MN can typically be exposed. Neurostimulation can aid in confirmation.

The present findings provide anatomical reference values that may facilitate identification of the MN during facial reanimation procedures. In clinical practice, however, these measurements should be interpreted in the context of individual variation in craniofacial dimensions, ethnicity, and age, particularly in pediatric patients. Consequently, intraoperative identification of the MN should continue to rely on anatomical landmarks in conjunction with careful dissection.

Direct visualization of skeletal landmarks is not feasible intraoperatively, as the dissection is performed at a more superficial plane. However, the zygomatic arch and the mandibular notch are easily palpable. Palpation of the mandibular notch can be facilitated by mobilizing the mandible, a maneuver we found particularly useful when the MN was obscured by dense fibers or adipose tissue. Surgeons should also be aware of the variable intramuscular branching pattern of the MN, which may facilitate selective nerve transfer while preserving residual masseter muscle function.

Our study analyzed 14 hemifaces from fresh-frozen adult cadavers, which may not fully reflect paediatric or variability across ethnic groups. In addition, the modest sample size limits statistical power, particularly for subgroup analyses such as sex-based comparisons, and may have obscured subtle anatomical differences. Nevertheless, our measurements align closely with prior studies in Caucasian and Asian populations.^12^^,^^18^^,^^20^ Future work, including radiologic assessment, may further validate these relationships in live patients and broaden applicability to pediatric cohorts.

Beyond anatomical localization, understanding the MN´s suitability as a donor nerve is essential. Its consistently high axonal load, favorable vector for smile elevation, and low donor-site morbidity have been well established. Multiple anatomic studies demonstrate substantial myelinated fiber counts,^3^^,^^12^ often exceeding those in CFNG. Additionally, because the masseter is typically innervated by multiple branches, harvesting a single branch rarely results in a significant functional deficit.^4^^,^^21^ Clinically, the MN also provides rapid and robust reinnervation, often within 3 – 6 months,^22^^,^^23^ which is advantageous compared with CFNG but must be weighed against the more limited spontaneity of movement and the potential for biting synkinesis.

A key strength of the present work is the precise definition of easily measurable anatomical reference points that facilitate reliable and efficient MN identification. Complemented by detailed illustrations and an instructional intraoperative video, our guide supports improved learning and standardization of the MN dissection technique. For novice surgeons, reproducible surface landmarks can increase confidence, reduce morbidity, and shorten operative time. For experienced surgeons, these data validate established norms and may aid in teaching, preoperative planning, and the development of procedural checklists.

## Conclusion

By establishing clear, easily measurable, and reproducible reference points, this study addresses a key challenge in dynamic facial reanimation: the reliable intraoperative identification of the masseteric nerve. Our findings refine existing anatomical landmarks, demonstrate consistency across cadaveric specimens, and translate directly into a safe and efficient dissection strategy. Integrating these guidelines into surgical practice may enhance outcomes, optimize operative workflow, and improve accessibility of masseteric nerve-based reanimation for both novice and experienced surgeons.

## Funding

This research received no specific grant from any funding agency in the public, commercial, or non-profit sectors.

## Ethical approval

Human cadavers were obtained through the Dutch body donation program for science and education in accordance with national legislation (Wet op de lijkbezorging, Art. 18 §1, Art. 19, Art. 67–70, April 1991, w.wetten.overheid.nl/BWBR0005009).

## Declaration of AI and AI-assisted technologies in the writing process

During the preparation of this work the authors used ChatGPT (OpenAI, San Francisco, USA) and Grammarly (Superhuman Platform Inc., San Francisco, USA) in order to improve phrasing and style of the manuscript text. After using this tool/service, the authors reviewed and edited the content as needed and take(s) full responsibility for the content of the publication.

## Credit statement

All photos and illustrations were created by Theresia Stigger, MD, specifically for this study. Intraoperative video footage was recorded by Koen Ingels, MD, and Niels van Heerbeek, MD, PhD. All images and video materials are original and have not been previously published.

**Video 1)** A video showing the intraoperative step-by-step identification of the masseteric nerve.

**Table 1)** A Table showing the topographical measurements of the masseteric nerve relative to key surface landmarks.

## Declaration of competing interest

The authors have no conflict of interest to declare with respect to the research, authorship, and/or publication of this article.

## Appendix Supplementary materials

Image, video 1 dummy alt text

## References

[1] Banks C.A., Jowett N., Iacolucci C., Heiser A., Hadlock T.A. (2019). Five-year experience with fifth-to-seventh nerve transfer for smile. Plast Reconstr Surg.

[2] Cotrufo S., Hart A., Payne A.P., Sjogren A., Lorenzo A., Morley S. (2011). Topographic anatomy of the nerve to masseter: an anatomical and clinical study. J Plast Reconstr Aesthet Surg.

[3] Coombs C.J., Ek E.W., Wu T., Cleland H., Leung M.K. (2009). Masseteric-facial nerve coaptation–an alternative technique for facial nerve reinnervation. J Plast Reconstr Aesthet Surg.

[4] Biglioli F., Frigerio A., Colombo V. (2012). Masseteric-facial nerve anastomosis for early facial reanimation. J Craniomaxillofac Surg.

[5] Spira M. (1978). Anastomosis of masseteric nerve to lower division of facial nerve for correction of lower facial paralysis. Preliminary report. Plast Reconstr Surg.

[6] Hontanilla B., Gómez-Ruiz R. (2010). Masseter nerve as "baby sitter" procedure in short-term facial paralysis. Eur J Plast Surg.

[7] Faria J.C., Scopel G.P., Ferreira M.C. (2010). Facial reanimation with masseteric nerve: babysitter or permanent procedure? Preliminary results. Ann Plast Surg.

[8] Sforza C., Ulaj E., Gibelli D.M. (2018). Three-dimensional superimposition for patients with facial palsy: an innovative method for assessing the success of facial reanimation procedures. Br J Oral Maxillofac Surg.

[9] Vachata P., Brusakova S., Lodin J., Sames M. (2019). Masseteric nerve supercharge bypass in primary reconstruction of facial nerve. Acta Neurochir.

[10] Kofler B., Ingels K. (2021). [Dynamic procedures for facial nerve reconstruction]. Laryngorhinootologie.

[11] Stigger T., Pulzl P., Wolfram D., Hofauer B.G., Magnato R., Kofler B. (2025). [Reconstruction and Reanimation of the Periorbital Region in Facial Paralysis]. Laryngorhinootologie.

[12] Borschel G.H., Kawamura D.H., Kasukurthi R., Hunter D.A., Zuker R.M., Woo A.S. (2012). The motor nerve to the masseter muscle: an anatomic and histomorphometric study to facilitate its use in facial reanimation. J Plast Reconstr Aesthet Surg.

[13] Collar R.M., Byrne P.J., Boahene K.D.O. (2013). The subzygomatic triangle: rapid, minimally invasive identification of the masseteric nerve for facial reanimation. Plast Reconstr Surg.

[14] Cheng A., Audolfsson T., Rodriguez-Lorenzo A., Wong C., Rozen S. (2013). A reliable anatomic approach for identification of the masseteric nerve. J Plast Reconstr Aesthet Surg.

[15] Caillouey A., Bettoni J., Olivetto M., Dakpé S., Testelin S. (2022). Masseteric nerve position on the "temporomandibular joint-chin tip" artificial axis: an anatomical study. Surg Radiol Anat.

[16] Pitanguy I., Ramos A.S. (1966). The frontal branch of the facial nerve: the importance of its variations in face lifting. Plast Reconstr Surg.

[17] Trussler A.P., Stephan P., Hatef D., Schaverien M., Meade R., Barton F.E. (2010). The frontal branch of the facial nerve across the zygomatic arch: anatomical relevance of the high-SMAS technique. Plast Reconstr Surg.

[18] Abdelkarim A., Allevi F., Bolognesi F. (2023). Intra-surgical optimized identification of masseteric nerve for central facial nerve neurorrhaphy: a retrospective study. J Craniomaxillofac Surg.

[19] Biglioli F., Guerra M.B., Rabbiosi D., Ciardiello C., Allevi F. (2021). Comparison of results utilizing one-step and two-step triple innervation techniques. J Craniomaxillofac Surg.

[20] Angspatt A., Pannanusorn C. (2018). The masseteric nerve: an anatomical study in Thai population with an emphasis on its use in facial reanimation. Asian J Surg.

[21] Brenner E., Schoeller T. (1998). Masseteric nerve: a possible donor for facial nerve anastomosis?. Clin Anat.

[22] Hontanilla B., Marre D., Cabello A. (2014). Masseteric nerve for reanimation of the smile in short-term facial paralysis. Br J Oral Maxillofac Surg.

[23] Eisenhardt S.U., Thiele J.R., Stark G.B., Bannasch H. (Aug 2013). [Comparison of cross face nerve graft with masseteric nerve as donor nerves for free functional muscle transfers in facial reanimation surgery]. Handchir Mikrochir Plast Chir.

